# Spectrum of false positivity for the fourth generation human immunodeficiency virus diagnostic tests

**DOI:** 10.1186/s12981-015-0086-3

**Published:** 2016-01-05

**Authors:** Peter Liu, Patrick Jackson, Nathan Shaw, Scott Heysell

**Affiliations:** Department of Internal Medicine, University of Virginia, 1215 Lee Street, Charlottesville, VA 22908 USA; Division of Infectious Diseases and International Health, University of Virginia, 345 Crispell Drive, Carter Harrison Building, Room 1523, Charlottesville, VA 22908 USA; University of Virginia School of Medicine, 1215 Lee Street, Charlottesville, VA 22908 USA

**Keywords:** HIV, Fourth generation assays, False positive

## Abstract

Novel fourth generation screening and confirmatory human immunodeficiency virus (HIV) assays are now commercially available and incorporated into new diagnostic algorithms. We report two cases involving a total of three patients which highlight the spectrum of false positivity for both the Abbott Architect p24 antigen/antibody assay and the confirmatory Multispot antibody differentiation test. We then discuss the mechanisms for false positivity and the associated clinical conditions or laboratory scenarios that may predispose to inaccurate interpretation.

## Background

HIV continues to pose a significant worldwide burden of morbidity and mortality. The advent of anti-retroviral therapy (ART) led to a paradigm shift of care and markedly improved outcomes in patients with HIV. Nevertheless, the global prevalence of HIV remains staggeringly high, and was estimated at 35.3 million in 2012 [1]. Additionally, despite multi-disciplinary advances in prevention and treatment algorithms, nearly 40,000 new cases of HIV are reported annually in the United States [2]. Thus, timely detection of HIV infection, particularly when viremia and transmissibility are high, is of considerable importance in continued efforts in both treatment and prevention of new infections. Novel fourth generation screening and confirmatory assays are now commercially available and incorporated into new diagnostic algorithms [3]. We report two cases involving a total of three patients which highlight the spectrum of false positivity despite the introduction of these assays, and review the mechanisms and associations with false positivity that remain critical for the clinician’s interpretation.

## Case 1

A 33 year old man presented with tender lateral neck swelling, cramping abdominal pain, and night sweats. He reported intra-nasal cocaine use and an instance of unprotected heterosexual contact in the preceding month. He denied intravenous drug use. On admission, he was afebrile with normal vital signs. His physical examination was significant for bulky adenopathy with submental, submandibular, cervical, inguinal, and axillary involvement. He also exhibited abdominal distention with hepatosplenomegaly.

The initial laboratory examination was remarkable for a white blood cell count of 18,760 with 54 % lymphocytes, platelet count of 123,000, aspartate aminotransferase (AST) of 51 U/L, and alanine aminotransferase (ALT) of 65 U/L. Haptoglobin was undetectable and lactate dehydrogenase was 743 U/L. HIV testing was performed singly and reactive by the Abbott Architect HIV antigen/antibody 4th generation screening assay (Abbott Laboratories, Abbott Park, IL, USA). Confirmatory testing using the Multispot HIV-1 and HIV-2 antibody differentiation assay (Bio-Rad Laboratories, Redmond, WA, USA), however, was nonreactive and an HIV viral load was undetectable. He had Epstein-Barr virus (EBV) viremia with 4580 copies/mL detected. Computed tomography (CT) imaging of his neck demonstrated numerous bilateral non-necrotic lymph nodes within the jugular chain, the largest of which measured 1.6 cm. CT of his abdomen and pelvis revealed extensive bulky adenopathy throughout both the peritoneal and retroperitoneal spaces. An excisional biopsy was also performed on a submental lymph node which demonstrated florid follicular hyperplasia with interfollicular immunoblastic proliferation, hilar plasmacytosis, and an atypical T cell population, with some features of an angioimmunoblastic T-cell lymphoma. He had concurrent laboratory abnormalities that included positive rheumatoid factor, direct antiglobulin test, and hepatitis B IgM. Hepatitis B viral load was undetectable. A CD4 count was 1042 cells/µL but represented only 10 % of T lymphocytes. Peripheral flow cytometry was performed with findings consistent with multiple T-cell phenotypic abnormalities, which were thought to represent a lymphoproliferative process versus polyclonal reactive process due to immune dysfunction.

After a multi-disciplinary review, the pathology of the biopsied lymph node was felt to represent reactive changes with atypical lymphocytes secondary to EBV infection without clear evidence of a malignant lymphoproliferative disorder. He was seen on several occasions in follow-up, and ultimately returned to his previous state of health. CT imaging revealed spontaneous resolution of lymphadenopathy. EBV viral load became undetectable and platelet counts normalized. While the patient’s age and previous multiple sexual partners made acute EBV infection less likely upon initial presentation, no alternative diagnosis was found.

## Case 2

A 52 year old woman presented with 3 weeks of jaundice and increasing abdominal distension. The initial laboratory examination was remarkable for a white blood cell count of 24,000 with an absolute lymphocyte count of 0, hemoglobin of 5.6 g/dL, platelet count of 83,000, and an international normalized ratio of 2.7 in the absence of anticoagulant medications. Her metabolic panel revealed a sodium of 124 mmol/L, total bilirubin of 4.2 mg/dL, and a mild increase in AST at 72 U/L.

A paracentesis was performed by the admitting physician. In the course of this procedure, the physician sustained a cut from a scalpel visibly soiled with patient blood. Per facility protocol, testing for blood-borne pathogens was performed on the patient. Hepatitis B and C serologies were nonreactive. HIV testing was performed singly using the Abbott Architect p24 antigen/antibody assay which resulted in positive. Confirmatory testing by Multispot antibody differentiation was positive for HIV-1 antibodies and negative for HIV-2 antibodies. On further interview, the patient denied a prior history of HIV testing. She denied previous blood transfusions, intravenous drug use, or tattoos. She endorsed two lifetime male sexual partners with inconsistent condom use.

Her initial blood sample arrived to the laboratory with two different patient labels, so HIV testing was repeated on the same day with a different venipuncture using the Abbott Architect p24 antigen/antibody assay and the same results returned. Additional testing revealed an absolute CD4 count of 116 cells/µL corresponding to 61 % of her T lymphocytes and a CD4/CD8 ratio of 2.18. Potentially confounding conditions were evaluated yielding a negative urine human chorionic gonadotropin, negative anti-nuclear antibody, and negative toxoplasma IgG and IgM. An HIV viral load showed no detectable RNA and a qualitative proviral DNA test was also negative. A Western blot was sent and returned as indeterminate due to nonspecific background reactivity.

Antiretroviral therapy was not initiated. CT of her abdomen showed innumerable peripherally enhancing and centrally necrotic hepatic lesions consistent with malignancy as well as enlarged retroperitoneal lymph nodes and spinal lesions consistent with metastases. A CEA and CA 19-9 were grossly elevated. The patient developed hemodynamically significant hematochezia requiring resuscitation with multiple units of blood products. She subsequently became hypoxic and hypotensive and required intubation with pressor support. After discussion with her family, support was withdrawn, the patient died, and the family declined autopsy.

The physician who was exposed to blood was started on post-exposure prophylaxis with emtricitabine-tenofovir and raltegravir. His baseline Abbott Architect p24 HIV antigen/antibody test (Abbott Laboratories, Abbott Park, IL, USA) performed singly on the same day as the aforementioned patient was reactive. As with the patient, the confirmatory Multispot antibody differentiation test which was drawn from a separate blood sample was also positive for HIV-1 but not HIV-2. An HIV viral load and qualitative proviral DNA test were both negative. He continued post-exposure prophylaxis for 1 week and then discontinued these medications. Follow up testing 6 months after exposure was negative on both HIV antigen/antibody test and proviral DNA.

In further review of specimen handling and processing, the patient and physicians blood samples were not shipped to the facility laboratory together as they had distinct origins from the medical intensive care unit and emergency department respectively. Due to concerns for mislabeling of the patients initial blood sample, repeat testing was performed with a separate venipuncture which yielded the same results. Given the congruent laboratory results from separate blood samples, no corrective action reports were filed from the laboratory on that day.

## Discussion

We report the cautionary spectrum of false positive HIV diagnostics in three patients (Table 1). In case one, a false positive fourth generation test was not confirmed by antibody differentiation or by nucleic acid amplification testing, representing a common scenario often observed with prior generations of HIV diagnostics. Yet, in case two, we report one likely and one confirmed false positive result which occurred with both the antigen/antibody assay and with a confirmatory HIV antibody differentiation test.

**Table 1:** Summary of diagnostic test results and proposed mechanisms of false positivity

	Abbott p24 antigen/antibody	MultiSpot HIV-1/HIV-2 differentiation	HIV proviral DNA	HIV RNA viral load	Alternative diagnosis/mechanism of false positivity	Other explanatory studies/reports
Case 1	Positive	Non-reactive	Not done	Negative	EBV infection and reactive lymphocytosis	[7–13]
Case 2 patient	Positive	Reactive to HIV-1	Negative	Negative	Metastatic cancer	[18]
Case 2 exposed health care provider	Positive	Reactive to HIV-1	Negative	Negative	Healthy/query poor specificity of individual test kit	[16]
Case 2 exposed health care provider (6 month follow up)	Negative		Negative	Negative		

Following HIV transmission, markers of infection can be detected by diagnostic assays in a predictable fashion. An ‘eclipse phase’ typically lasting 10–14 days is characterized by infection in local mucosal and lymphoreticular tissue during which systemic viremia is not detected. Approximately 7 days later, viral RNA becomes present in serum at quantities detectable by nucleic acid amplification. Capsid protein p24 is the next chronological measurable component of acute infection by way of detection of soluble antigen. Approximately 5 days after detection of p24 antigen, seroconversion occurs and anti-HIV antibody becomes evident by reactive results to various available ELISA assays [4, 5].

Various commercial fourth generation tests are available for use, all of which utilize the combined detection of p24 antigen and HIV-1/HIV-2 antibody. Ly et al. performed a large analysis to investigate the sensitivity and specificity of various fourth generation tests. Numerous assays including the Abbott Architect p24 HIV antigen/antibody test (Abbott Laboratories, Abbott Park, IL, USA) were able to detect infection prior to p24 antigen assays and in fewer than 3 days after nucleic acid testing while maintaining a specificity of greater than 99 % [6]. Fourth generation tests can effectively shorten the time frame in which HIV infection is undetectable prior to seroconversion. While reported sensitivity and specificity of fourth generation tests remain higher than 99 % in numerous studies, several instances of discordance between screening and confirmatory techniques have been described [1, 7–10].

Lee et al. performed a comparative analysis of commercially available 3rd and 4th generation screening assays in effort to evaluate the specificity and false positivity of the newer 4th generation assay [11]. Four seroconversion panels were included for analysis including a batch of serum samples previously known to have produced discordant results between screening and confirmatory testing. Of the 54 known false positive serum samples, all 54 demonstrated continued discordance with 3rd generation testing, while 34 instances were reported with the 4th generation assay. Patients from whom discordant samples were obtained also had various concurrent clinical conditions including rheumatologic diseases, viral hepatitis, malignancy, infection with *Mycobacterium tuberculosis*, a history of multiple pregnancies, and recent *Rickettsia* infection, conditions which have been similarly associated with false positive screening tests in other studies [12–14]. Shida et al. describe a patient with a reactive 4th generation HIV diagnostic secondary to an angioimmunoblastic T-cell lymphoma [15], the lymphoproliferative disease originally suspected in “Case 1”. Various other phenomena were concurrently reported to cause false positive results including autoimmune hemolytic anemia, high anti-nuclear antibody titers, and a polyclonal hypergammaglobulinemia [15]. Importantly for roll-out of diagnostics for other HIV endemic countries, discordance between serologic screening tests and confirmatory assays have also been demonstrated in the setting of elevated IgG antibodies to *Schistosoma* species in a study of adolescents in Tanzania [16].

The new Multispot antibody differentiation test has been reported to have sensitivity and specificity greater than 99 % [17], comparing favorably with traditional Western blotting as a confirmatory assay [18]. Yet as Case 2 demonstrates, false positive results have been reported, more commonly with HTLV-I, HTLV-II, toxoplasmosis, and SLE [19]. It is biologically plausible that conditions which would yield a false-positive 4th generation antigen/antibody assay could similarly cause a false positive antibody differentiation assay. Remarkably however, in Case 2, the false positive screening and confirmatory testing was observed not only for a patient with a likely malignancy but also for the healthcare worker with no known complicating health problems. This suggests that the etiology of the erroneous result may not have been intrinsic to the patient and, indeed, may have been secondary to reduced specificity of the batch of testing kits [17].

In 2014, the centers for disease control and prevention (CDC) updated recommendations for the diagnosis of HIV infection to include a novel algorithm using the fourth generation screening and confirmatory assays [3]. Initial screening should begin with a combination immunoassay or fourth generation test that utilizes detection of both HIV-1/HIV-2 antibodies with HIV-1 p24 antigen. Negative results conclude testing, while reactive results necessitate further testing with a HIV-1/HIV-2 antibody differentiation assay. Specimens that demonstrate reactivity on the initial screening immunoassay, but negative or indeterminate results on antibody differentiation assay, should undergo nucleic acid testing. In this report, the CDC algorithm guided the clinicians to the correct identification of a false positive test in Case 1. In the second case, on the other hand, the algorithm would have led to two incorrect HIV diagnoses with the potential for substantial harm.

## Conclusion

Rapid HIV diagnostics such as fourth generation antigen/antibody assays and HIV antibody differentiation assays permit the identification of increased numbers of recent HIV infections and can help facilitate faster entry into care. While these testing modalities have high reported sensitivity and specificity, like all tests, they remain imperfect. CDC guidelines have been issued to assist clinicians in the interpretation of these results, but these cases emphasize that correct use of the algorithm continues to require careful clinical judgment.

## Abbreviations

HIV: human immunodeficiency virus
ART: anti-retroviral therapy
AST: aspartate aminotransferase
ALT: alanine aminotransferase
CT: computed tomography
EBV: epstein-barr virus
CDC: centers for disease control and prevention
HTLV: human T-lymphotropic virus
SLE: systemic lupus erythematosus
